# Efficacy of hypertonic dextrose proliferation therapy in the treatment of rotator cuff lesions: a meta-analysis

**DOI:** 10.1186/s13018-024-04754-4

**Published:** 2024-05-15

**Authors:** Ting Zhang, YanFu Wang, Lin Ding, ChaoYang Ma

**Affiliations:** Department of Rehabilitation Medicine, Central Hospital of Wuhan, Hubei Province, China

**Keywords:** Hypertonic dextrose, Prolotherapy, Rotator cuff lesions, Tendon, Meta-analysis

## Abstract

**Background:**

One of the most prevalent illnesses of the shoulder is rotator cuff tendinosis, which is also a major contributor to shoulder discomfort and shoulder joint dysfunction. According to statistics, rotator cuff tendinosis occurs in 0.3–5.5% of cases and affects 0.5–7.4% of people annually. It will be necessary to conduct a meta-analysis to evaluate the efficacy of hypertonic glucose proliferation therapy in the treatment of rotator cuff problems.

**Methods:**

The databases Cochrane PubMed, Library, Web of Science and EMbase, are retrieved by the computer. Individuals with rotator cuff lesions in the intervention group were treated with hypertonic dextrose proliferation therapy, whereas individuals in the control condition were treated with a placebo. Outcome markers for rotator cuff lesions patients; Pursuant to studies, the visual analogue scale (VAS) score, the shoulder pain & disability index (SPADI), & other metrics are used to evaluate the effects of hypertonic dextrose proliferation treatment on individuals with rotator cuff diseases. After carefully evaluating the calibre of the literature, data analysis was performed utilising the RevMan 5.3 programme.

**Results:**

Meta-analysis finally contained 6 papers. In six investigations, the test & control group’s VAS scores improved, with the test team’s score considerably outperforming the control team [standardized mean difference (SMD): 1.10; 95% Cl: 0.37,1.83; *P* < 0.01], shoulder pain and disability index (SPADI) score (SMD:8.13; 95% Cl: 5.34,10.91; *P* < 0.01), Flexion (SMD:5.73; 95% Cl: 0.99,10.47; *P* < 0.05), Abduction (SMD:6.49; 95% Cl: 0.66,12.31; *P* < 0.05), Internal rotation (SMD:-1.74; 95% Cl: -4.25,0.78; *P* = 0.176) and External rotation (SMD:2.78; 95% Cl: -0.13,5.69; *P* = 0.062).

**Conclusion:**

The findings of this study suggest that individuals with rotator cuff injuries may benefit from hypertonic dextrose proliferation treatment based on the visual analogue scale (VAS) score, the Shoulder Pain and Disability Index (SPADI) score, Flexion, & Abduction. These results must, nevertheless, be supported by high-caliber follow-up research.

## Introduction

Rotator cuff tendinosis, as one of the most prevalent conditions affecting the shoulder joint, is a significant contributor, etc [1]. . According to reports, the yearly prevalence of rotator cuff tendinosis is 0.5–7.4% and the incidence rate is 0.3–5.5% [2]. In 1972, Neer described rotator cuff tendon disease as three progressive stages: acute tendinitis, tendon degeneration or partial rupture, and complete rupture [3]. Daniel et al. defined rotator cuff tendinosis as a painful disease caused by tendon degeneration or partial tear [4]. Lewis believed that the underlying mechanism is tendon overuse, repair disorders, and ultimately impaired mobility [5]. A variety of conditions may contribute to shoulder cuff tendon disease, including specifically internal, external, & comprehensive factors. The compression of tendons caused by surrounding bone and soft tissue structures is an external factor, while older age, damage to nutrient vessels, and excessive use of tendons are internal factors. These factors can lead to tendon wear and partial or full layer tearing of the shoulder sleeve [6]. Tendons, a kind of connective tissue, are crucial for the movement of the body because they link muscles to bones. Tendon disease can occur in any tendon, but the most common ones are the shoulders, elbows, knees, and Achilles tendons. The occurrence of tendinosis is usually related to overuse or injury of tendons. Common symptoms include pain, swelling, limited mobility, and decreased muscle strength. Tendonopathy can be divided into many types, including tendinitis (inflammation of the tendon), Tendinopathy (degeneration or degeneration of tendon) and tendon rupture. The pathological characteristics of this disease are characterized by chronic changes such as collagen fiber degeneration disorder, cell hypertrophy, and tendon thickening visible under the microscope. The treatment methods for tendinosis include rest, physical therapy, medication, and surgery. Rest can reduce the stress and inflammatory response of tendons. Physical therapy includes massage, physical therapy, and tendon traction, which helps promote blood circulation and tendon repair. The commonly used drugs include nonsteroidal anti-inflammatory drugs, local hormone injections and Analgesics. Severe tendon disease may require surgical repair of the tendon.

Prolotherapy originated in the 5th century before christ. Hippocrates proposed to simulate tendon healing through stimulation to achieve the effect of repairing damaged tendons, which is called proliferation and regeneration therapy [7]. It was found that secondary inflammation in the affected area not only did not worsen the condition but also promoted the self-healing effect of locally damaged connective tissue. The term Prolotherapy means offspring, which induce the regeneration of new cells by stimulating or damaging the injured site. At present, prolotherapy has been widely used in clinical practice. Non-operative treatment is selected for patients with traumatic diseases of the Skeleton, such as temporomandibular joint disorder, Neuropathic pain, pain caused by intervertebral disc herniation, low back pain, lumbar sprain, lumbar muscle strain, pain in sacroiliac joints, and diseases related to bone joints, tendons and ligaments of lower limbs, such as knee Osteoarthritis, secondary ankle sprain, Achilles tendinitis Non-stop Achilles tendinitis, etc., so Prolotherapy is a pain management method. The injected proliferative agents are clinically divided into irritants, chemoattractants and penetrants, among which the more common clinical drugs are phenol solution, Zinc sulfate solution, glycerin, sodium Cod liver oil and hypertonic dextrose solution.

Prolotherapy is a method of injecting proliferating agents into the damaged tendon or ligament to induce new cells to proliferate and repair soft tissue. Hypertonic dextrose is commonly used as the proliferating agent. Hypertonic dextrose can induce hyperosmotic dehydration at the injection site, induce inflammatory reactions, increase glucose utilization, & encourage type III collagen fibres at the damage site to become type I collagen fibres, promoting repair. Hypertonic dextrose Prolotherapy can induce inflammatory reactions in damaged tissues and initiate body repair [8, 9].

Unfortunately, although many studies have evaluated the efficacy of hypertonic glucose proliferation therapy in the treatment of rotator cuff problems, there have been no relevant meta-analyses that integrate the latest research.In this study, we conducted a meta-analysis to assess the efficacy of hypertonic dextrose proliferation therapy in the treatment of rotator cuff problems, which will bring light for those who suffered from rotator cuff lesions.

## Materials and methods

### Study selection

Design of the randomized controlled trial(RCT) studies that have been published on the outcomes of hypertonic dextrose proliferation treatment in individuals with rotator cuff lesions. Nevertheless, animal experimentation was excluded. Prolotherapy, hypertonic dextrose, & rotator cuff are the search terms. The time frame for the search was from the opening of the library through February 2023. There are total 191 literatures in the study, including PubMed (*n* = 40),Embase(*n* = 69),Cochrane Library(*n* = 28), Web of Science (*n* = 54). Records after duplicates removed (*n* = 102), abstracts screened (*n* = 18) ,records excluded(*n* = 7). There are total 6 studies included in quantitative synthesis.

### Participants selection

#### Inclusion criteria

Patients who meet the diagnostic criteria for Rotator Cuff Lesions patients, have poor oral drug efficacy, and a strong willingness to receive injection therapy. Among them, patients with non traumatic and refractory rotator cuff diseases such as chronic rotator cuff injury, long head tendon disease of the biceps brachii, partial tears, and full thickness tears, and calcific tendonitis are all included in the list of rotator cuff disease patients.

#### Exclusion criteria

(1) Joint infection (including local skin infection), joint tumor or tuberculosis in the affected joint; (2) Abnormal coagulation function; (3) Existence of severe cardiovascular diseases and severe liver and kidney dysfunction; (4) Combining cognitive and mental disorders, unable to cooperate with treatment and follow-up.

### Interventions types

Individuals with rotator cuff lesions in the intervention group were treated with hypertonic dextrose proliferation therapy, whereas individuals in the control condition were treated with a placebo. The placebo treatment mainly uses 5% physiological saline, and the control group’s treatment also includes conventional treatment methods such as physical therapy and exercise therapy.

### Outcome measure types

Outcome markers for rotator cuff lesions patients; Pursuant to studies, the visual analogue scale (VAS) score, the shoulder pain & disability index (SPADI), & other metrics are used to evaluate the effects of hypertonic dextrose proliferation treatment on individuals with rotator cuff diseases. Flexion (3), abduction (4), internal rotation (5), and external rotation (6). Using at least one of the aforementioned scales, the literature that was analysed for this study assessed the outcome measures.

### Search strategy

The databases Cochrane PubMed, Library, Web of Science and EMbase, are retrieved by the computer. To do a literary search, you must first hunt for relevant papers in English databases, then utilise a mix of topic words & keywords to narrow down your search results, & last use “MeSH Terms” to pinpoint the subject terms.

Extraction of data & Assessment of Quality:

After the first review of the abstract, two researchers independently conducted the procedure of literature screening. The entire text was then read in order to gain the results of the literary screening. Researchers may contrast screening results, discuss contradictory literature, or get in touch with a third researcher until the results are agreed upon. Among the data that were extracted are fundamental details about the literature, research kind, study object, intervention content, sample size, outcome measures, etc.

### Statistical analysis

This meta-analysis was performed using Review Manager (RevMan). The standardised mean difference (SMD) & 95%Letters to the Zone (confidence interval, CI) are employed as a measure of impact. (2) To determine if there is heterogeneity among research, chi-square tests are performed; if *P* > 0.1, I2<50%, and the included studies were considered to be more homogenous, then there was less heterogeneity. It is appropriate to perform a fixed-effects model meta analysis if *P* < 0.1, I2 > = 50%, and heterogeneity were found in the encompassed research. Investigate diverse sources, Meta analyses using a random-effects model are done when there is no clinical heterogeneity. Subgroup studies of possible differences in qualitative traits were also carried out.

## Results

### Search results

**Fig. 1:**
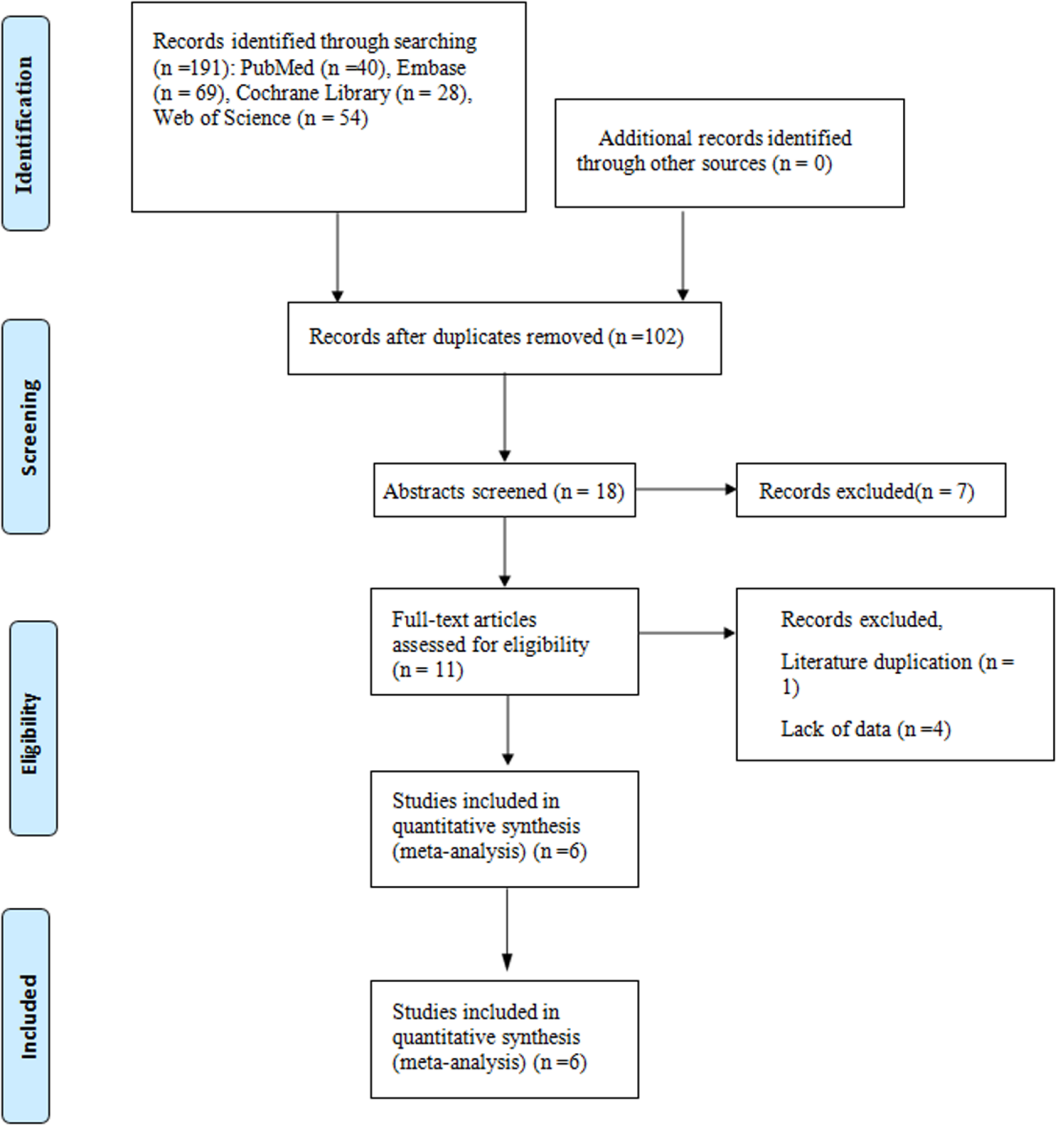
Flow chart

The search approach led to the discovery of 191 references. After duplicate studies were identified, the abstract and title of 18 papers were scanned. Following that, 11 articles’ complete texts were evaluated. Following a thorough text examination, 5 records were eliminated due to the following factors: duplication of literature (*n* = 1) & information deficiency (*n* = 4). In the end, six studies [10–15] were included in our meta-analysis (Table 1). The PRISMA statement flow chart (Fig. 1) shows this process.

**Table 1:** The basic characteristics of the included studies

Study(ref.)	Research	Sample Size(T/C)	Man/Woman	Age (years)(T/C)	T	C	Main Outcomes
Bertrand,2016	RCT	27/20	30/17	53.8±13.5/51.1±9.2	Three monthly Hypertonic Dextrose+programmed physical therapy	Three monthly Placebo (saline)+programmed physical therapy	①
Lin,2022	RCT	29/28	30/27	49.10±8.44/52.18±9.83	20% Hypertonic Dextrose	Placebo (5% normal saline)	①②③④⑤⑥
Lin,2019	RCT	16/15	19/12	46.25±5.69/48.6±5.95	20% Hypertonic Dextrose	Placebo (5% normal saline)	①②③④⑤⑥
Sari,2020	RCT	30/30	None	52.11±10.78	20% Hypertonic Dextrose	Placebo (5% normal saline)	①
Lee,2015	case-control study	57/53	40/70	54.1±7.8/55.8±6.6	16.5% Hypertonic Dextrose	conservative treatment	①②③④⑤⑥
Seven,2017	RCT	57/44	None	50.19±12.13/46.31±10.6	Hypertonic Dextrose+home exercise program	prolotherapy injection+home exercise program	①②③④⑤⑥

T: trial group; C: control group. ① The visual analog scale (VAS) score; ② The shoulder pain and disability index (SPADI) score; ③ Flexion; ④ Abduction; ⑤ Internal rotation; ⑥ External rotation

### The visual analog scale (VAS) score

**Fig. 2:**
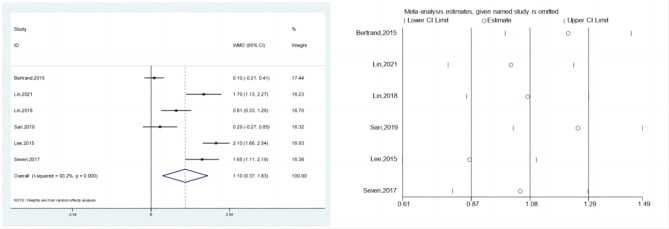
Forest illustration and ssensitivity analysis of the visual analog scale (VAS) score

Six investigations reported the VAS scores for the test & control categories. In accordance with meta-analysis, the test category’s VAS score improvement was appreciably bigger than that of the control category’s (SMD: 1.10; 95% Cl: 0.37,1.83; *P* < 0.01, Fig. 2). A sensitivity analysis was conducted (Fig. 2) due to the high degree of variability in the results of each of these experiments. When treating individuals with rotator cuff lesions, hypertonic dextrose proliferation therapy improves VAS score more than the control category. The Begg’s Test and Egger’s Test readings for the present research were 0.707 & 0.249, correspondingly. These results are highly steady, as there is no overt publication bias.

### The shoulder pain and disability index (SPADI) score

**Fig. 3:**
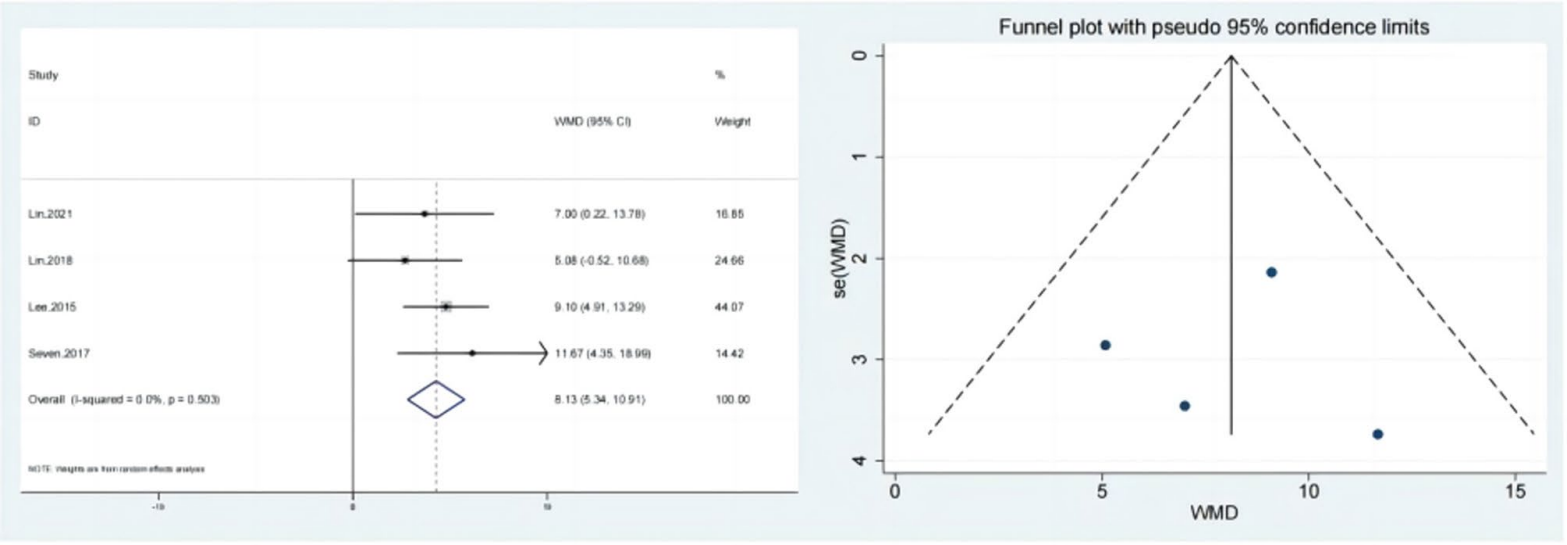
Forest illustration and funnel plot of the Shoulder Pain and Disability Index (SPADI) score

**Fig. 4:**
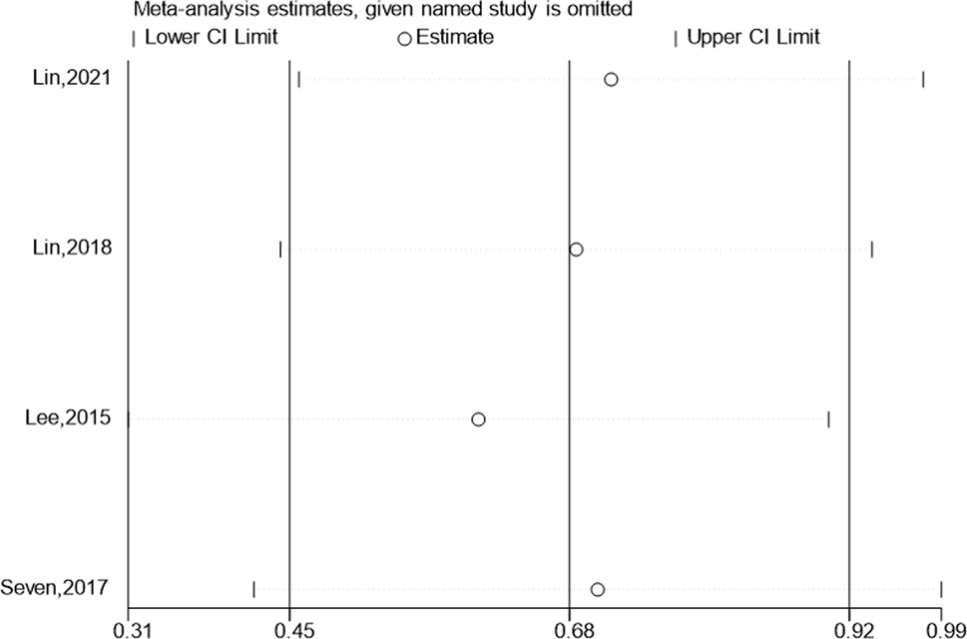
Sensitivity analysis of the Shoulder Pain and Disability Index (SPADI) score

The SPADI results for the test group & the control team were published in 4 studies. The SPADI score gain was substantially bigger than that of the control category, based on meta-analysis. (SMD:8.13; 95% Cl: 5.34,10.91; *P* < 0.01, Fig. 3). Figure 3 shows a funnel plot that is largely symmetrical. The results of all of these trials showed minor heterogeneity, hence a sensitivity analysis was carried out (Fig. 4). When treating individuals with rotator cuff problems, hypertonic dextrose proliferation therapy improves SPADI score more than the control group. The results of the present research’s results passed the Begg’s Test & Egger’s Test with scores of 0.734 & 0.980, accordingly, indicating that there is no obvious publication bias.

### Flexion

**Fig. 5:**
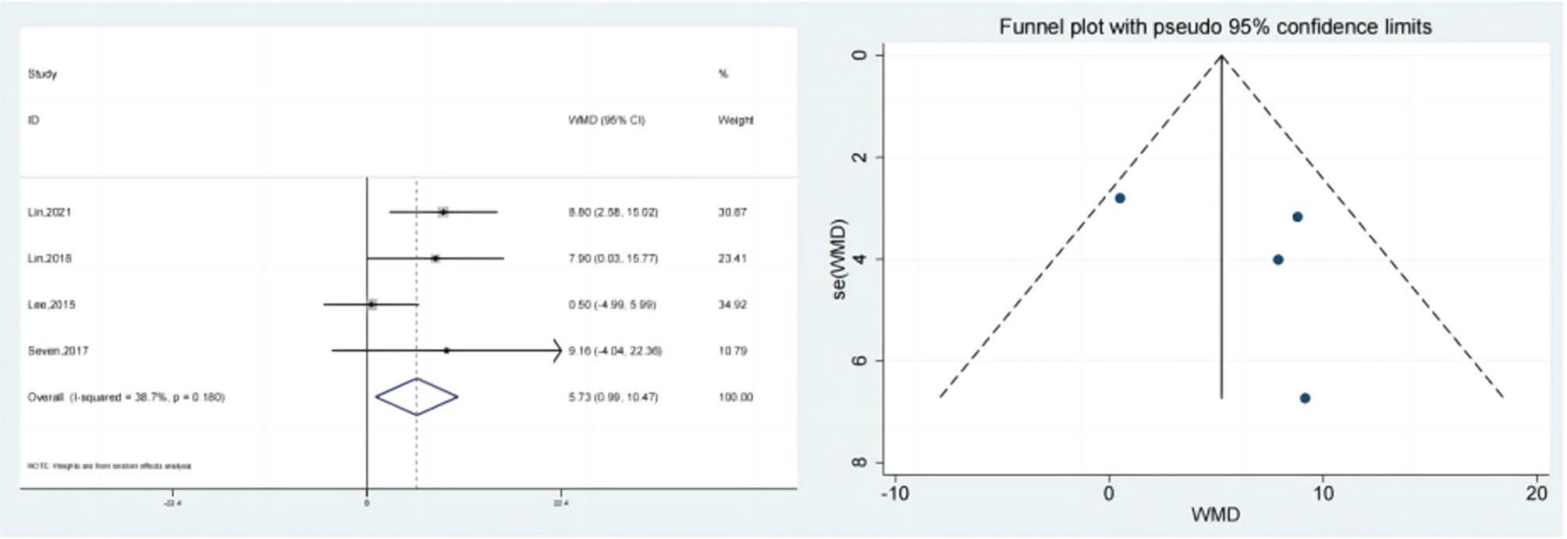
Forest illustration and funnel plot of the Flexion

**Fig. 6:**
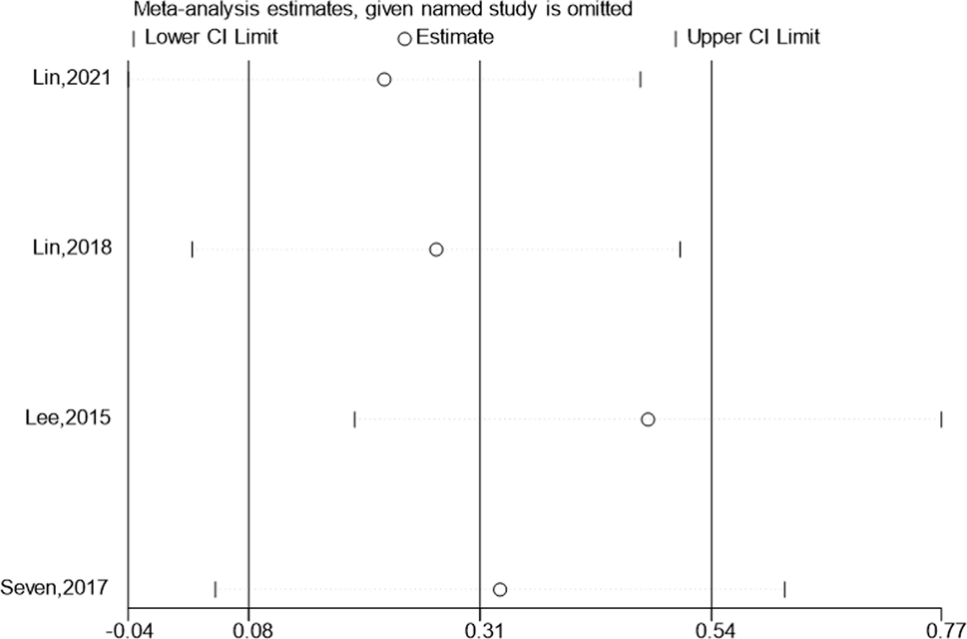
Sensitivity analysis of the Flexion

Four studies reported the test & control category’s flexion. As per meta-analysis, Flexion considerably outperformed the control category in terms of improvement (SMD:5.73; 95% Cl: 0.99,10.47; *P* < 0.05, Fig. 5). The funnel plot (Fig. 5) is largely symmetrical. An analysis of the trials’ findings’ moderate heterogeneity was done, & the results are shown in Fig. 6. Individuals with rotator cuff lesions are treated with hypertonic dextrose proliferation therapy, which improves Flexion more than the control condition. The values of the Begg’s Test and Egger’s Test, which are 1.000 & 0.465, accordingly, indicate that the results of the present investigation are quite consistent as well as that there is no overt publication bias.

### Abduction

**Fig. 7:**
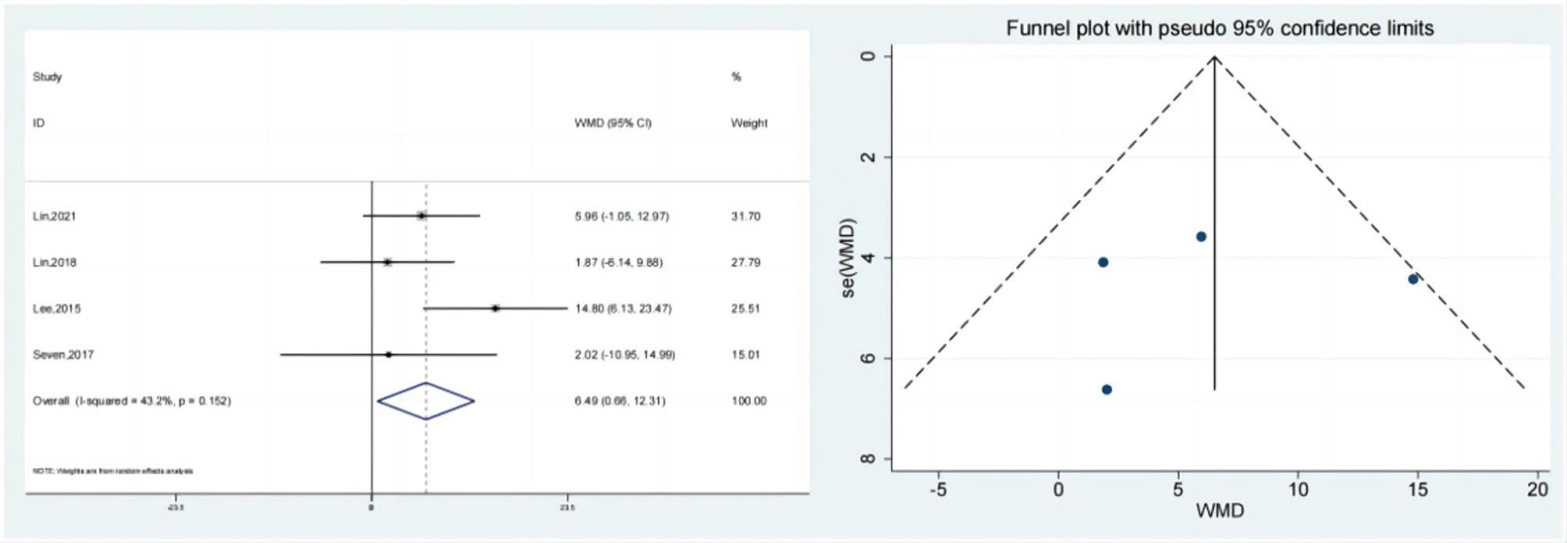
Forest illustration and funnel plot of the Abduction

**Fig. 8:**
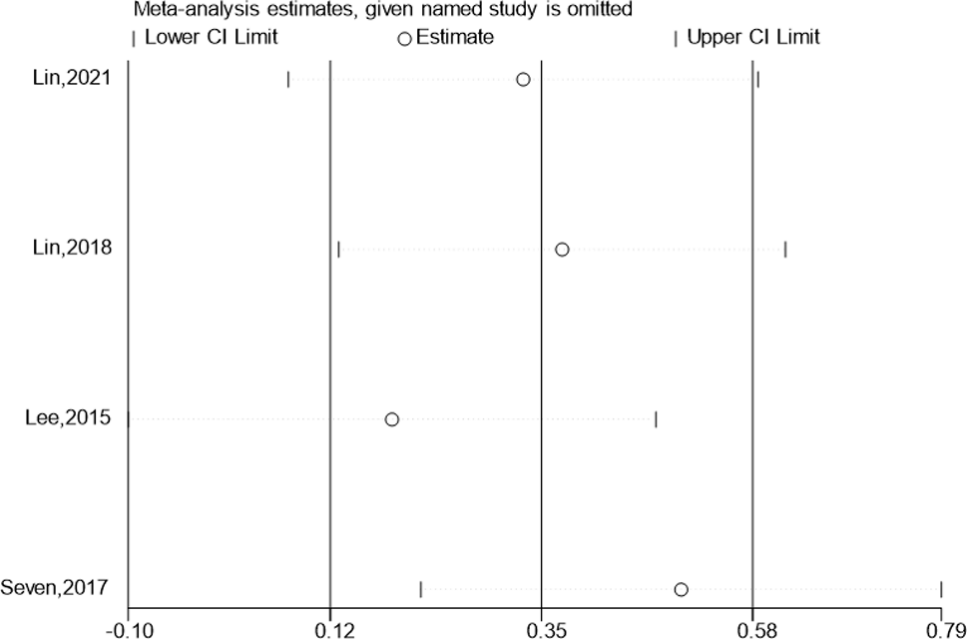
Sensitivity analysis of the Abduction

The abduction of the test & control category was documented in 4 research. Meta-analysis revealed that Abduction considerably outperformed the control category in terms of progress (SMD:6.49; 95% Cl: 0.66,12.31; *P* < 0.05, Fig. 7). Funnel plot (Fig. 7) is largely symmetrical. An analysis of the trials’ findings’ moderate heterogeneity was done, & the results are shown in Fig. 8. When treating individuals with rotator cuff diseases, hypertonic dextrose proliferation therapy leads to a greater improvement in abduction than the control category. The values of the Begg’s Test and Egger’s Test, which are 1.000 & 0.898, accordingly, indicate that the findings of the present research are generally consistent and that there is no discernible publication bias.

### Internal rotation

**Fig. 9:**
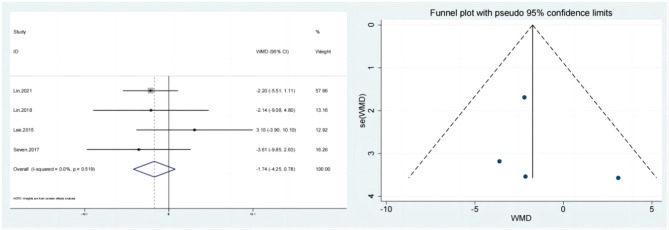
Forest illustration and ffunnel plot of the Internal rotation

**Fig. 10:**
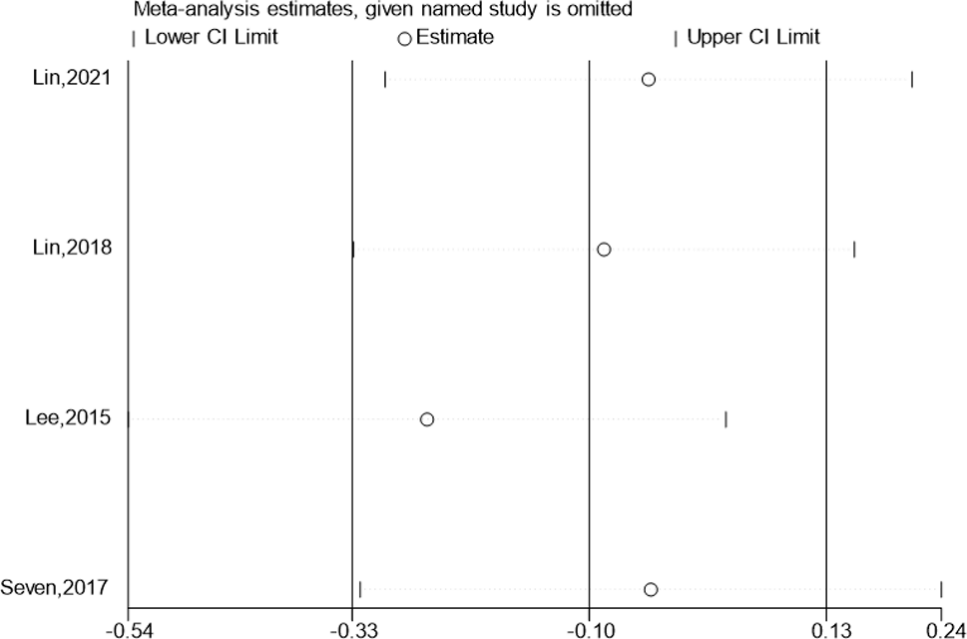
Sensitivity analysis of the Internal rotation

Internal rotation of the test & control category was observed in 4 studies. No statistically significant distinction among the internal rotation improvement group and the control group was found by meta-analysis. (Fig. 9; SMD:-1.74; 95% Cl: -4.25,0.78; *P* = 0.176). The funnel plot (Fig. 9) is largely symmetrical. An analysis of the trials’ findings’ moderate heterogeneity was done, & the results are shown in Fig. 10. When used to treat individuals with rotator cuff lesions, hypertonic dextrose proliferation therapy did not result in a greater improvement of internal rotation than the control category. The values of the Begg’s Test and Egger’s Test, which are 0.308 & 0.625, accordingly, indicate that the results of this study are reasonably consistent as well as that there is no discernible publication bias.

### External rotation

**Fig. 11:**
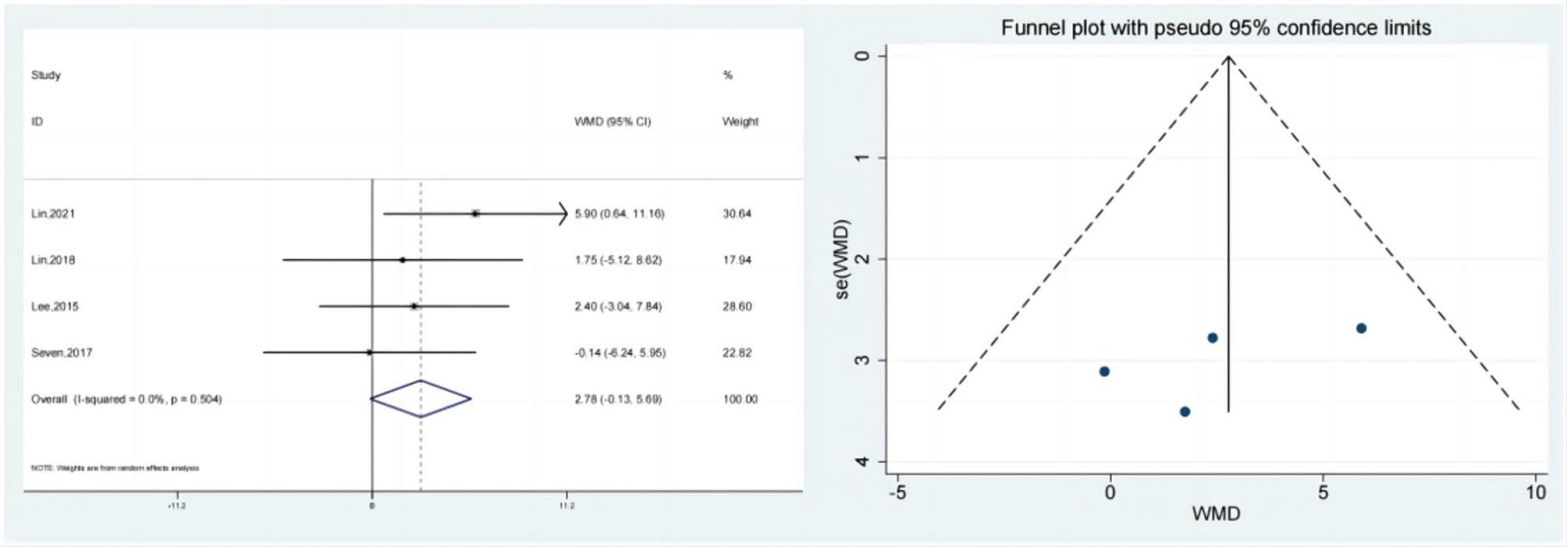
Forest illustration and funnel plot of the External rotation

**Fig. 12:**
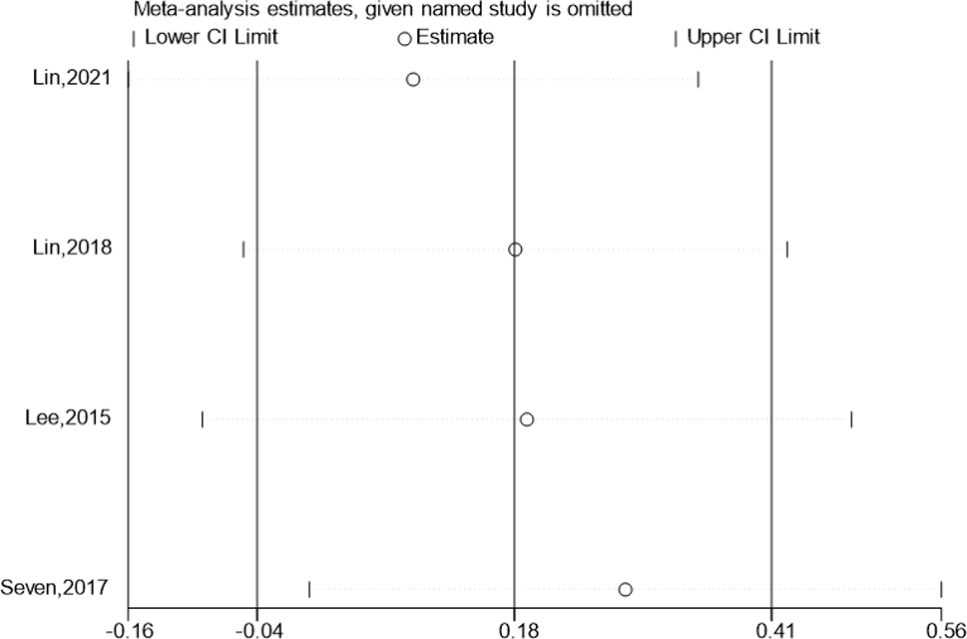
Sensitivity analysis of the External rotation

It was reported in 4 research where the test & control category were externally rotated. (SMD: 2.78; 95% Cl: -0.13,5.69; *P* = 0.062, Fig. 11) Meta-analysis revealed that the external rotation improvement was not statistically dissimilar from control category (Fig. 11), the funnel plot is largely symmetrical. A sensitivity analysis was carried out (Fig. 12) when it was discovered that the outcomes of all of these studies exhibited considerable heterogeneity. Hypertonic dextrose proliferation therapy did not result in a greater improvement of external rotation in individuals with rotator cuff injuries compared to the control category. Pursuant to the findings of the Begg’s & Egger’s tests, both of which have values of 0.308 & 0.352, the outcomes of this research are extremely consistent, and there isn’t any obvious publication bias.

## Discussion

The most important findings of this meta-analysis were that hypertonic dextrose proliferation therapy may be beneficial to individuals with rotator cuff lesions. There were 216 people in the experimental group and 190 people in the control group, for a total of 6 studies. According to the meta-analysis, individuals with rotator cuff injuries who had hypertonic dextrose proliferation treatment demonstrated significant VAS score improvement compared to controls. The experimental category’s VAS score improved in a decent manner, as per meta-analysis. As per SPADI score meta-analysis’s findings, the test category’s SPADI score improvement was noticeably more than control category. According to the Flexion meta-analysis findings, the test category’s Flexion improvement was noticeably more than control category when compared to the control category. According to the findings of the meta-analysis of abduction, as compared to the control category, the test category’s abduction performance considerably improved. Meta-analysis revealed that the test category’s Internal rotation & External rotation had no statistically significant difference from the control category when compared to the findings of the internal and external rotation meta-analysis. There was no noticeable publication bias, as seen by the symmetrical funnel plots for all analyses.

Hypertrophic therapy is a treatment method that stimulates the repair of soft tissues (including tendons, ligaments, and articular cartilage) in the body to treat pain and enhance the strength of soft tissues. Hyperplasia therapy is simple and safe. Injecting the medicinal solution into the damaged tissue locally produces an inflammatory response, induces a proliferative response, and thus reactivates the body’s repair mechanism. Hypertrophic therapy is equivalent to giving the body tissues a chance to repair again, which can improve discomfort symptoms and even eliminate pain. The commonly used proliferative agents currently include high concentrations of glucose water, platelet rich plasma, etc. Mechanism of prolotherapy is not very clear. It may be that hyperplasia fluid produces local inflammatory reaction, increases local growth factor release, promotes collagen healing, and reduces pain [16]. Inflammatory reaction is one of hypertonic glucose’s primary modes of action, and there is evidence that it is useful in treating painful tendinosis [17]. Inflammatory reaction can induce the release of growth factors, promote fibrosis, and also reduce inappropriate Angiogenesis and accompanying nerve growth, thus alleviating pain and repairing tendons.

Hypertonic dextrose mainly plays a role in the treatment of tendinosis from the inflammatory stage, which leads to infiltration of white blood cells and macrophages, initiation of wound healing cascade reaction, and proliferation of muscle and leg fibers through such channels as “osmotic shock” of muscle and leg cells, mutual induction and trend of inflammatory transmitters, or increase of Antigenicity of host cells [18]. After inflammation begins, granulocytes and macrophages are lured to the injection site of hypertonic dextrose, and fibroblasts deposit new collagen under its lure [19]. The new collagen produced through this process contracts and pulls the ligaments tighter. While specific mechanism of action of this treatment is currently unclear and there is no consensus. According to research, the injected proliferative osmotic agent is essentially congruent with the body’s normal healing process. A hypertonic dextrose injection increases the synthesis of extracellular matrix, strengthening ligaments, muscular legs, & joints, also improves their longevity and functioning [20].

According to a study, an Achilles tendon disease model in rats showed [21], the size, strength, and intensity of the Achilles tendon increased after 2 weeks of intervention with hypertonic glucose compared to before the intervention. Tumour necrosis factor-α (TNF-α) was discovered by Eliasson et al. to be involved in the rat Achilles tendon disease model’s healing process [22]. Pires et al. found that in animal tendon injury models, tumor necrosis factor α The expression increased from 2 h after the initial use of hypertonic glucose intervention to 9 days, and gradually decreased after 2 weeks [23]. Although these experiments are more limited to animals, they may also provide explanations for the mechanisms of action in the human body. These clinical and experimental studies show that hypertonic dextrose Prolotherapy with different concentrations can effectively alleviate tendinosis.

This article summarizes research on the efficacy of hypertonic glucose proliferation therapy in the treatment of rotator cuff problems, and has certain clinical and therapeutic significance. There are also limations for the search. First, it was limited to English; no literature in other languages was found. Furthermore, there could have been biassed selection and insufficient studies incorporated. As a result, you ought to evaluate some of this meta analysis’ conclusions with objectivity.

## Conclusion

According to the visual analogue scale (VAS) score, the Shoulder Pain as well as Disability Index (SPADI) score, Flexion, & Abduction, the results of this study indicate that hypertonic dextrose proliferation therapy may be beneficial to individuals with rotator cuff lesions. However, these findings need to be confirmed by additional studies of excellent quality.

## Data Availability

The data could be obtained by contacting the corresponding author.
